# A nomogram for predicting left atrial thrombus or spontaneous echo contrast in non-valvular atrial fibrillation patients using hemodynamic parameters from transthoracic echocardiography

**DOI:** 10.3389/fcvm.2024.1337853

**Published:** 2024-02-08

**Authors:** Decai Zeng, Xiaofeng Zhang, Shuai Chang, Yanfen Zhong, Yongzhi Cai, Tongtong Huang, Ji Wu

**Affiliations:** Department of Ultrasound, First Affiliated Hospital, Guangxi Medical University, Nanning, China

**Keywords:** non-valvular atrial fibrillation, nomogram, spontaneous echo contrast (SEC), thrombus, hemodynamic parameters

## Abstract

**Background:**

Atrial fibrillation (AF) is the most common cardiac arrhythmia and is associated with a high risk of stroke. This study was designed to investigate the relationship between hemodynamic parameters and left atrial thrombus/spontaneous echo contrast (LAT/SEC) in non-valvular atrial fibrillation (NVAF) patients and establish a predictive nomogram that integrates hemodynamic parameters with clinical predictors to predict the risk of LAT/SEC.

**Methods:**

From January 2019 to September 2022, a total of 354 consecutive patients with NVAF were enrolled in this cross-sectional study at the First Affiliated Hospital of Guangxi Medical University. To identify the optimal predictive features, we employed least absolute shrinkage and selection operator (LASSO) regression. A multivariate logistic regression model was subsequently constructed, and the results were visualized with a nomogram. We evaluated the model's performance using discrimination, calibration, and the concordance index (C-index).

**Results:**

We observed a 38.7% incidence of SEC/TH in NVAF patients. Independent influencing factors of LAT/SEC were identified through LASSO and multivariate logistic regression. Finally, four indicators were included, namely, previous stroke/transient ischaemic attack (OR = 4.25, 95% CI = 1.57–12.23, *P* = 0.006), left atrial volume index (LAVI) (OR = 1.04, 95% CI = 1.01–1.06, *P* = 0.001), S/D ratio (OR = 0.27, 95% CI = 0.11–0.59, *P* = 0.002), and left atrial acceleration factor (OR = 4.95, 95% CI = 2.05–12.79, *P* = 0.001). The nomogram, which incorporated these four influencing factors, demonstrated excellent predictive ability. The training set had a C-index of 0.878, while the validation set had a C-index of 0.872. Additionally, the calibration curve demonstrated great consistency between the predicted probabilities and the observed outcomes, and the decision curve analysis confirmed the important clinical advantage of the model for patients with NVAF.

**Conclusion:**

Our findings indicate that an enlarged left atrium and abnormal hemodynamic parameters in the left atrial and pulmonary veins are linked to a greater risk of LAT/SEC. Previous stroke/transient ischaemic attack, LAVI, the S/D ratio, and left atrial acceleration factor were independently associated with LAT/SEC in NVAF patients. With the incorporation of these four variables, the developed nomogram effectively predicts the risk of LAT/SEC and outperforms the CHA_2_DS_2_-VASc score.

## 1 Introduction

Atrial fibrillation (AF) is the most common arrhythmia encountered in clinical practice. Given the aging populations and the accumulation of predisposing factors, experts anticipate that the incidence of AF will increase at least a 2.5 folds by 2050 (1). The association between AF and stroke, along with various cardiovascular conditions, is well-documented. Moreover, the use of anticoagulant therapy has been demonstrated to significantly reduce the risk of stroke in individuals with AF and is instrumental in the prevention of recurrent strokes (2). Consequently, it is crucial to perform a comprehensive stroke risk assessment and to initiate anticoagulation therapy promptly, in order to effectively manage thromboembolic events and reduce mortality (3).

Various clinical conditions and biomarkers have been recognized as indicators of stroke outcomes in patients with Non-valvular Atrial Fibrillation (NVAF). Among these risk factors, left atrial thrombus (LAT) and spontaneous echocardiographic contrast (SEC) are widely recognized as significant contributors to cardiogenic embolism in NVAF (4). Although transesophageal echocardiography (TEE) remains the gold standard for detecting LAT and SEC, its invasive character and the need for conscious sedation limit the wide applications (5). Therefore, the development of a non-invasive and equally reliable diagnostic alternative would represent a substantial advancement in clinical practice.

Efforts to predict LAT/SEC have led to the creation of various nomograms that integrate clinical and demographic data, providing personalized risk assessments and guiding therapeutic decisions. The CHA_2_DS_2_-VASc score, which is widely used to stratify the risk of stroke in NVAF patients (6) has shown limitations in its correlation with LAT formation. Because thrombi may be detected in patients with low CHA_2_DS_2_-VASc scores (7). This underscores the need for a more precise model that includes relevant clinical and echocardiographic parameters. While previous research has primarily focused on left atrial size, specifically left atrial diameter, this approach is somewhat limited and imprecise (8, 9). Instead, incorporating detailed hemodynamic parameters could yield richer insights into the correlation between left atrial characteristics and stroke risk.

Recognizing the influence of changes in hemodynamics within the left atrium (LA) on the thrombus formation and subsequent increased risk of stroke (10), it becomes crucial to accurately assess the hemodynamic condition of the LA for preventive strategies. Establishment and validation a nomogram based on hemodynamic parameters has the potential to greatly improve efforts towards stroke prevention in NVAF patients.

In this study, we sought to address the following objectives: Firstly, to investigate the relationship between hemodynamic parameters and LAT/SEC, and secondly, to establish a nomogram based on hemodynamic parameters and clinical influencing factors for estimating the risk of LAT/SEC in NVAF patients.

## 2 Methods

### 2.1 Data source

Between January 2019 and September 2022, the First Affiliated Hospital of Guangxi Medical University recruited a cohort of 512 consecutive patients with NVAF who were eligible for radiofrequency ablation and/or occlusion of the left atrial appendage.

### 2.2 Participants

The research included individuals with paroxysmal or persistent AF. Paroxysmal AF was characterized by episodes that spontaneously reverted to a normal heart rhythm within a week, whereas persistent AF referred to cases that lasted longer than 7 days and required pharmacological treatment or electrical cardioversion to restore a normal heart rhythm.

**Figure 1:**
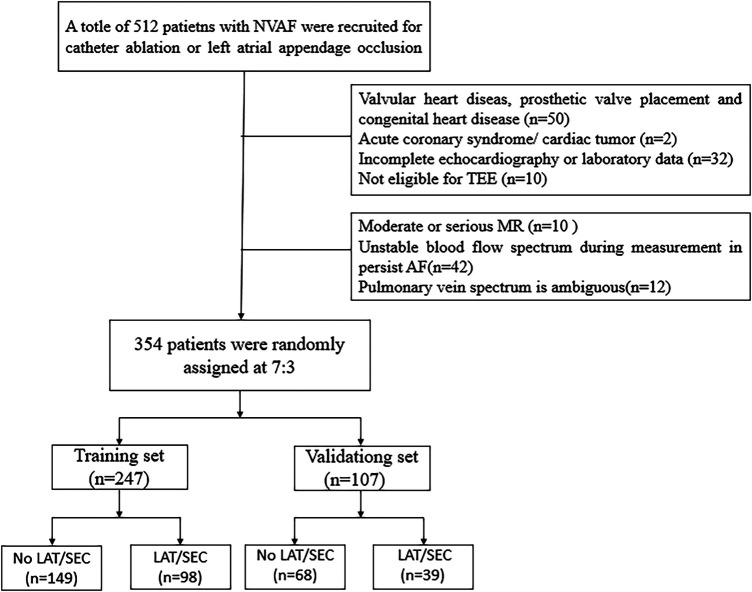
The flowchart of the selection of study participants. (NVAF, non-valvular atrial fibrillation; TEE, transesophageal echocardiography; MR, mitral regurgitation; LAT/SEC, left atrial thrombus/spontaneous echo contrast).

The exclusion criteria are as follows: (1) valvular heart disease, prosthetic valve replacements, and congenital heart disease; (2) moderate or severe mitral regurgitation (MR); (3) acute coronary syndrome; (4) Unstable blood flow spectrum during measurement in persist AF; (5) patients with indistinct pulmonary vein waveforms; and (6) cardiac tumors. According to inclusion and exclusion criteria, a total of 354 individuals were included and then randomized into two groups: the training cohort (*n* = 247) and the validation cohort (*n* = 107). The participant selection flowchart is depicted in Figure 1.

Ethical approval was obtained from the Ethics Committee of the First Affiliated Hospital of Guangxi Medical University (approval number 2022-KT-077). The research was conducted according to the Helsinki declaration guidelines and all study participants signed the informed consent form.

### 2.3 Definition of explanatory variables

Demographic, laboratory, and clinical data were retrieved from electronic medical records. Hypertension was defined as systemic arterial blood pressure with systolic measurements ≥140 mmHg and diastolic measurements ≥90 mmHg. To diagnose diabetes, fasting serum glucose levels of at least 7.0 mmol/L and/or random glucose levels of at least 11.1 mmol/L were required. Congestive heart failure diagnosed based on characteristic symptoms and subsequent confirmed by a physician's diagnosis.

### 2.4 Evaluation of CHA_2_DS_2_-VASc score and classification of risk

The CHA_2_DS_2_-VASc score is a clinical tool used to assess the risk of stroke in patients with NVAF. It includes components for congestive heart failure or left ventricular dysfunction (C), hypertension (H), the patient's age (A) with two points for age ≥75 years, or one point for ages 65–74, diabetes mellitus (D), a history of thromboembolism, transient ischemic attack (TIA), or stroke (S), vascular disease (V), and the sex category (Sc) with one point for females. The purpose of this score is to help determine the need for anticoagulant treatment, following the current recommendations which suggest its use in individuals with confirmed AF if they have a CHA_2_DS_2_-VASc Score of ≥2 for males or ≥3 for females.

### 2.5 Echocardiographic examination

**Figure 2:**
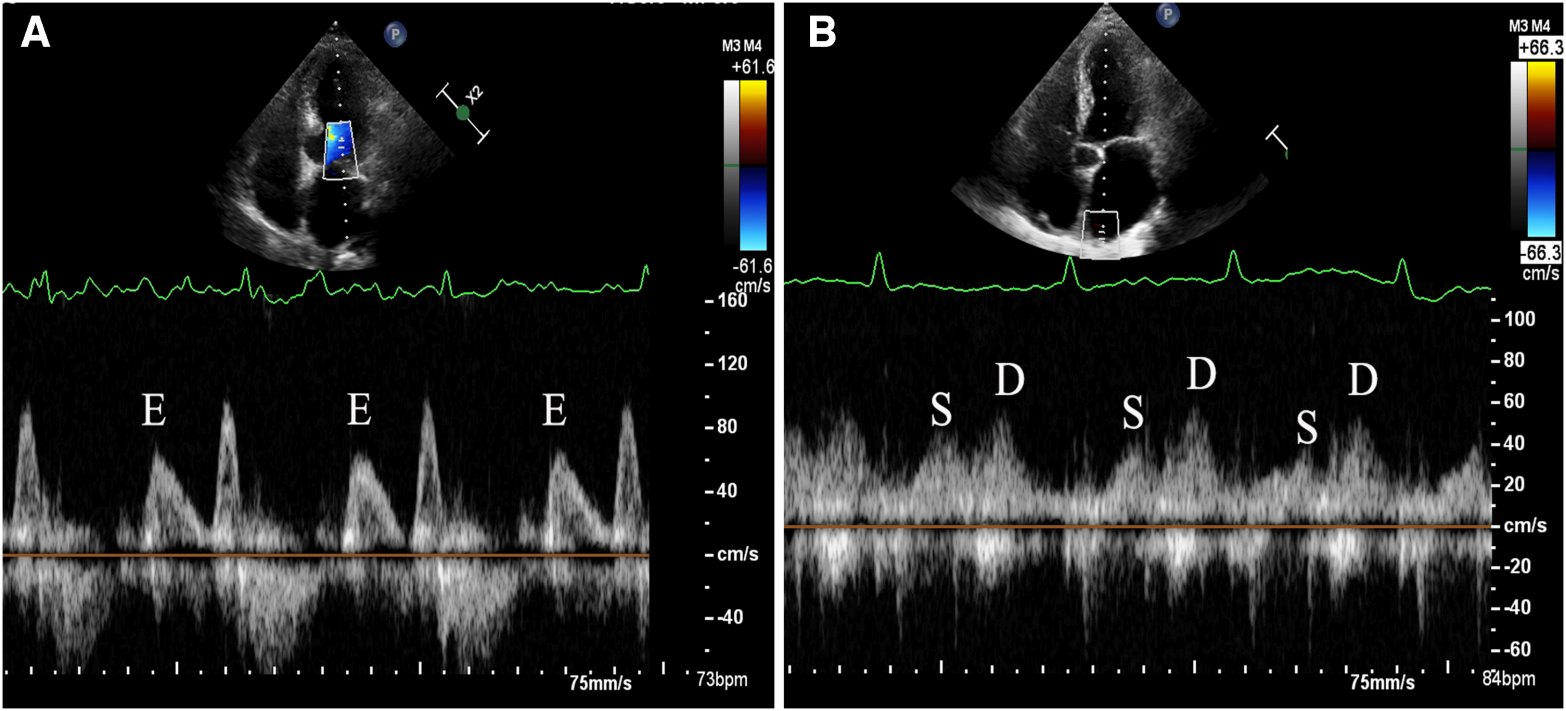
Demonstration of measurement method for left atrial acceleration factor (α) by transthoracic echocardiography. (**A**) The apical 4-chamber view was used to measure the peak velocities of the diastolic early (E) trans-mitral Doppler flow, placing the sample volume at the tip of the mitral leaflets. (**B**) The maximum inflow velocities of the LA were defined using the recorded velocity of the right upper pulmonary vein inflow during systole (S) and diastole (D).

Transthoracic echocardiography (TTE) was conducted using the commercially available EPIQ 7C system (Koninklijke Philips N.V., Netherlands). The S5-1 probe with a frequency range of 1–5 MHz was utilized for the TTE examination. For TEE, the X8-2t probe with a frequency range of 2–8 MHz was employed. The time gap between the TEE and TTE should not exceed 48 h to ensure consistency and reliability of the cardiac assessment findings. The standard method was used to measure the linear dimensions of the cardiac chambers. LV volumes and EFs were calculated using the modified Simpson's method, based on the apical 2 and 4 chamber views. The validated Devereux's formula was applied to calculate the left ventricular (LV) mass: LV mass = 0.8{1.04[(SWT + LVEDD + PWT)^3^−LVEDD^3^]} + 0.6, where SWT = left ventricular end-diastolic septal wall thickness, LVEDD = left ventricular end-diastolic diameter, and PWT = left ventricular end-diastolic posterior wall thickness. The biplane Simpson's method was used to measure the LA volume from the apical 2- and 4-chamber views. Afterwards, the LV mass and LA volume were normalized to the body surface area. The apical 4-chamber view was used to measure the peak velocities of the diastolic early (E) trans-mitral Doppler flow, placing the sample volume at the tip of the mitral leaflets. Tissue Doppler imaging was used to measure the e' velocity at the septal and lateral mitral annulus during peak early diastole. The E/e' ratio was then calculated by dividing E by the mean e’. The maximum inflow velocities of the LA were defined using the recorded velocity of the right upper pulmonary vein inflow during systole (S) and diastole (D), and the S/D ratio was calculated. When the patients were in AF, the echocardiographic variables, such as the trans-mitral Doppler flows, mitral annular velocity (e'), and flow in the right upper pulmonary vein, were measured during 5 consecutive cardiac cycles and subsequently averaged to obtain consistent and reliable data. When the patients were in sinus rhythm, all Doppler measurements were averaged from three measurements. The LA acceleration factor (α) (11) was deﬁned as the ratio of E to the average of S and D (Figure 2). The calculation formula is as follows: *α* = E/[(S + D)/2]. After excluding all contraindications for TEE examination, a detailed explanation of the procedure was provided, and written consent was obtained from all patients prior to undergoing the TEE examination. A thrombus within the LA was characterized as a well-defined, uniformly dense mass, distinct from the surrounding LA endocardium and pectinate muscles. It was observed in multiple imaging planes. Additionally, smoke-like echocardiographic findings with swirling blood flow in the LA and/or left atrial appendage, referred to as SEC, were identified even when gain settings were optimally adjusted. SEC is recognized as a marker of potential thromboembolic events, and is indicative of a hypercoagulable state. All measurements were carried out and interpreted by experienced physicians who were blinded to the objectives of the study to maintain an unbiased assessment.

### 2.6 Statistical analysis

R software (version 4.0) was utilized for all statistical analyses. A Student's *t*-test was used to compare continuous data with a normal distribution, and the results were reported as mean and standard deviation (SD). The Mann–Whitney test was employed to compare continuous variables with a non-normal distribution, and results are expressed as median with interquartile range. Categorical data were presented as frequencies and percentages and analyzed using either the *χ*^2^ test or Fisher's exact test. There were 29 variables included in the subjects. To select the best predictive features, the “glmnet” package was used for the least absolute shrinkage and selection operator (LASSO) analysis, and the numerical model was established using the “rms” package for multivariate logistic regression analysis. The selection of the optimal combination of influencing factors was performed using LASSO regression with ten-fold cross-validation. Subsequently, the chosen factors from LASSO regression were further examined through multiple forward step-wise logistic regression. A nomogram was created by incorporating the independent risk factors in the multivariate logistic regression analysis using the “regplot” package. The points for each independent variable were summed to obtain the total points, which were subsequentlyconverted to predicted probabilities. The predictive performance of the nomogram was assessed using the concordance index (C-index) and calibration with 500 bootstrap samples to minimize overfit bias. To evaluate the clinical usefulness of the nomogram at different threshold probabilities, decision curve analysis was conducted using the R library “rmda” package in the primary dataset.

## 3 Results

### 3.1 Characteristics of the study population

**Figure 3:**
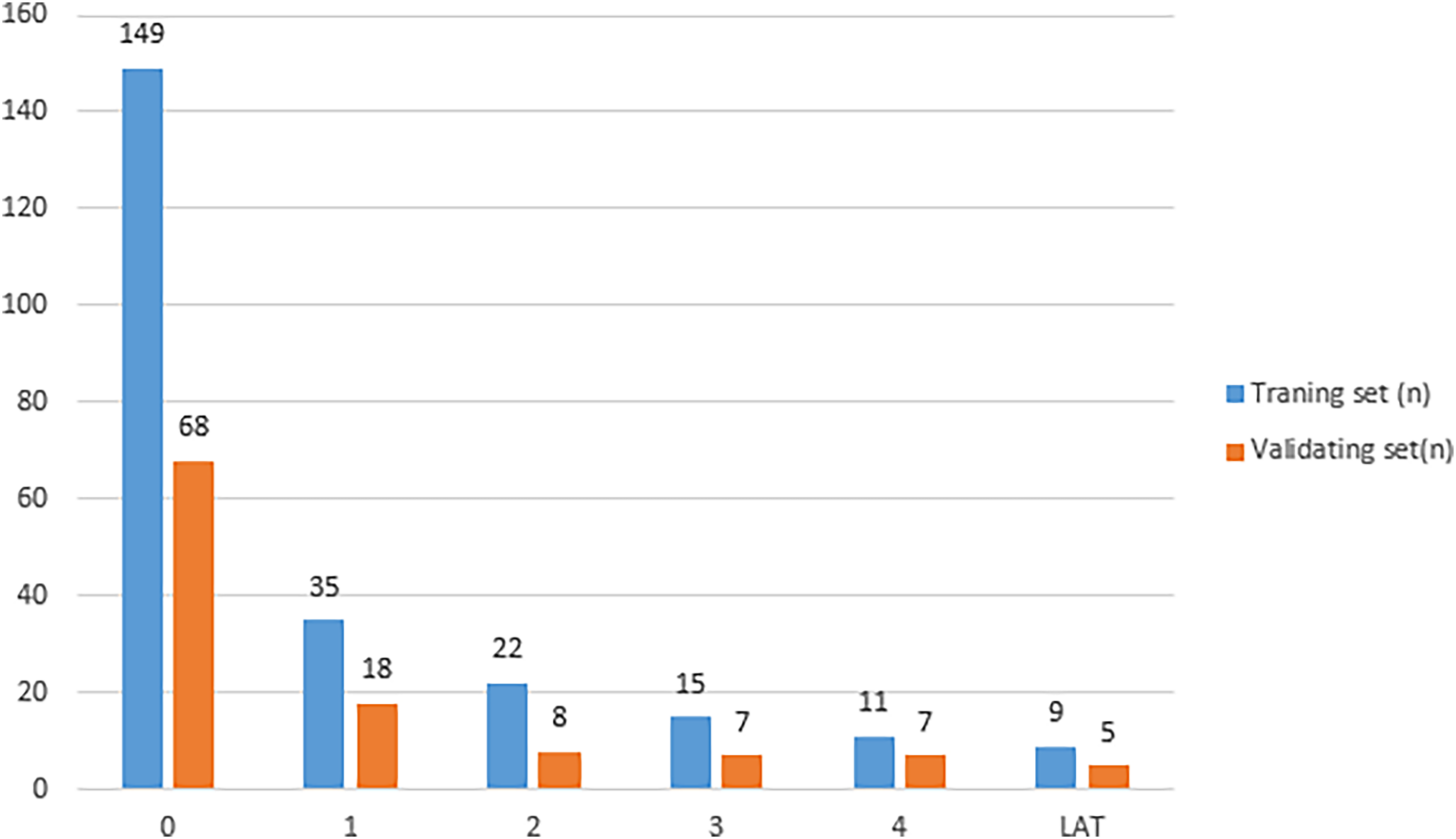
The distribution of subjects in different grades of LAT/SEC. (LAT/SEC, left atrial thrombus/spontaneous echo contrast).

In this study, 354 patients with NVAF were included. Of these, 247 patients (69.8%) were assigned to the training cohort and 107 patients (30.2%) were assigned to the validation cohort. No significant differences were observed between the training and validation cohorts in terms of their baseline information, indicating that the two groups were comparable. A total of 137 patients were identified with LAT/SEC. The distribution of subjects in different grades of SEC/LAT was shown in Figure 3. The training cohort included 98 patients (39.6%) with LAT/SEC, whereas the validation cohort included 39 patients (36.4%). The baseline characteristics of the patients stratiﬁed by LAT/SEC were outlined in Table 1.

**Table 1:** Baseline characteristics of the 354 patients.

Variable	Overall	No LAT/SEC	LAT/SEC	*P*-value
	(*n* = 354)	(*n* = 217)	(*n* = 137)	
Age, years	59.62 ± 10.85	57.6 ± 11.7	62.81 ± 8.45	<0.001
Female sex, *n* (%)	100 (28)	68 (31)	32 (23)	0.133
Body surface area, m²	1.72 ± 0.19	1.71 ± 0.18	1.75 ± 0.22	0.095
Systolic blood pressure, mmHg	128.21 ± 18.81	128.25 ± 18.78	128.14 ± 18.93	0.956
Diastolic blood pressure, mmHg	79.86 ± 11.9	78.78 ± 11.12	81.58 ± 12.89	0.037
Medical history				
Hypertension, *n* (%)	178 (50)	93 (43)	85 (62)	<0.001
Diabetes, *n* (%)	49 (14)	24 (11)	25 (18)	0.080
Dyslipidemia, *n* (%)	104 (29)	69 (32)	35 (26)	0.255
Previous stroke/TIA, *n* (%)	47 (13)	16 (7)	31 (23)	<0.001
Vascular disease, *n* (%)	147 (42)	81 (37)	66 (48)	0.057
Coronary heart disease, *n* (%)	65 (18)	34 (16)	31 (23)	0.132
History of heart failure, *n* (%)	20 (6)	5 (2)	15 (11)	0.001
Persistent AF, *n* (%)	150 (42)	62 (29)	88 (64)	<0.001
CHA_2_DS_2_ -VASc score	2 (1,4)	2 (1,3)	3 (2,4)	<0.001
Laboratory data				
Scr, µmol/L	84.36 ± 28.69	81.05 ± 19.5	89.6 ± 38.56	0.017
Ccr, ml/min	77.66 ± 17.87	81.43 ± 17.28	71.65 ± 17.19	<0.001
NT-proBNP, pg/ml	563.5 (155, 1,066)	284 (90, 948)	980 (562, 1,881)	<0.001
Troponin I, ng/L	4 (2, 9)	3 (2, 7)	7 (4, 14)	<0.001
Medication				
Antiplatelet, *n* (%)	43 (12)	23 (11)	20 (15)	0.340
Warfarin, *n* (%)	20 (6)	5 (2)	15 (11)	<0.001
NOAC, *n* (%)	230 (65)	137 (63)	93 (68)	0.425
Echocardiographic parameters				
LVEDV, ml	126.17 ± 35.59	121.08 ± 33.99	134.22 ± 36.69	<0.001
LVESV, ml	49.46 ± 29.34	44.81 ± 27.78	56.83 ± 30.31	<0.001
LV mass index, g/m^2^	131.68 ± 36.11	124.35 ± 33.82	143.28 ± 36.7	<0.001
LV ejection fraction, %	63.06 ± 10.85	65.42 ± 9.25	59.33 ± 12.1	<0.001
LAVI, ml/m^2^	50.59 ± 20.69	42.78 ± 18.21	62.96 ± 18.23	<0.001
E/e’	9.04 ± 3.46	8.33 ± 3.01	10.17 ± 3.82	<0.001
S/D	1.04 ± 0.56	1.22 ± 0.57	0.75 ± 0.39	<0.001
LA acceleration factor (α)	0.96 ± 0.42	0.8 ± 0.3	1.21 ± 0.46	<0.001

Values are mean ± SD, *n* (percentage), or median (25th, 75th percentile).

LAT/SEC, left atrial thrombus/spontaneous echocardiographic contrast; LAVI, left atrial volume index; LV, left ventricle; LVEDV, left ventricular end diastolic volume; LVESV, left ventricular end systolic volume.

### 3.2 Selected predictors

**Figure 4:**
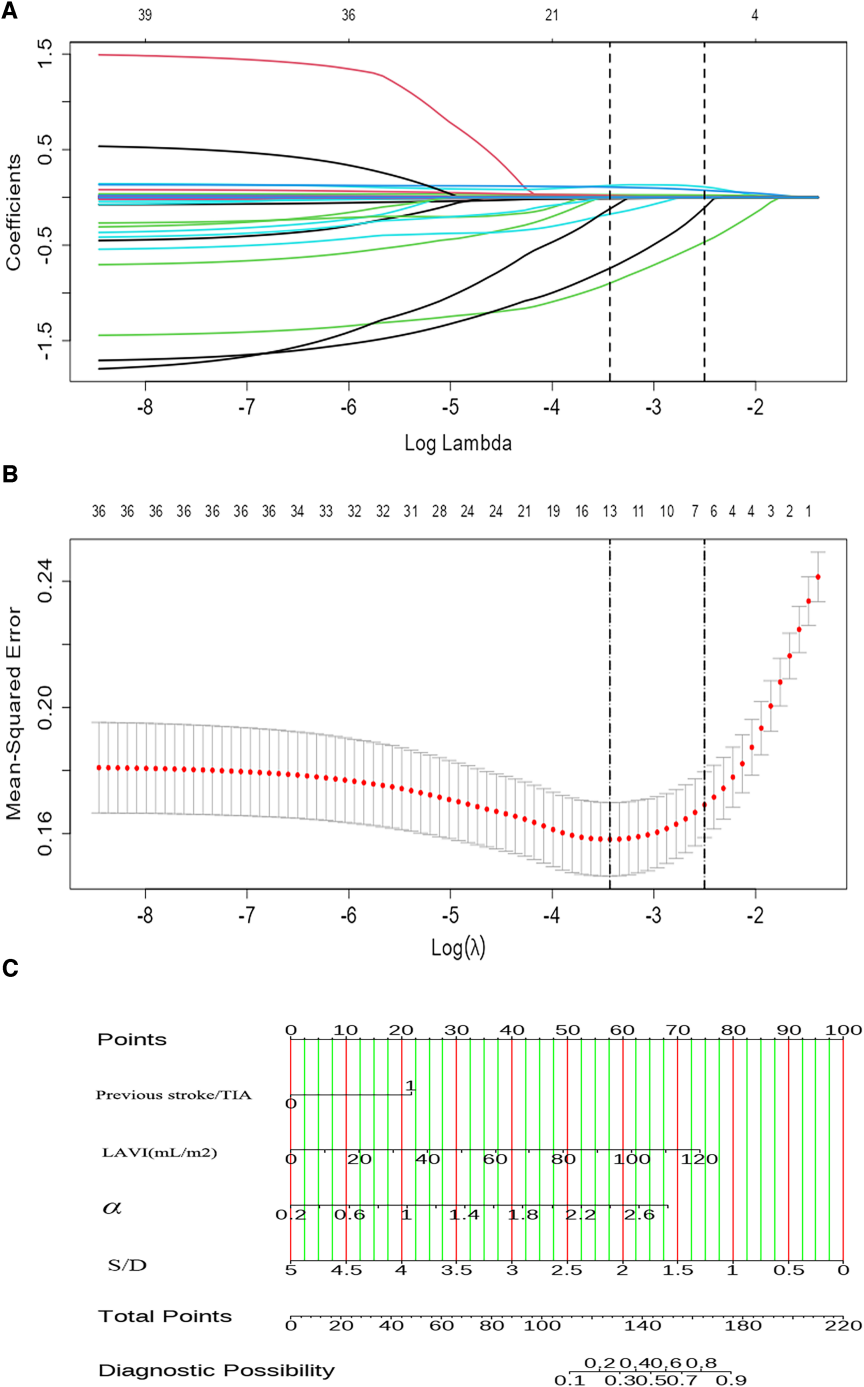
The LASSO COX regression is used for feature selection. (**A**) The tuning parameter (λ) in the regression is selected using 10-fold cross-validation and minimum criteria. A plot of the partial likelihood binomial deviance vs. log (λ) shows the optimal values where features are selected. Dotted vertical lines are set at these optimal values and also at one standard error of the minimum criteria. (**B**) The coefficient profiles for clinical features in the LASSO regression are plotted against a sequence of log (λ). A dotted vertical line is set at the nonzero coefficients selected using 10-fold cross-validation, which includes six nonzero coefficients. (**C**) A nomogram designed to estimate the risk of LAT/SEC in patients with NVAF.

LASSO regression analysis was applied to all variables, determining the optimal lambda parameterization via 10-fold cross-validation. Out of the 29 variables, six were identified as potential predictive features (Figures 4A,B). These included previous stroke/TIA, NT-proBNP, LAVI, LVEF, S/D ratio, and α. These six variables were then advanced to multivariate logistic regression analysis within the training cohort. Of these factors, previous stroke/TIA (OR = 4.25, 95% CI: 1.57–12.23, *P* = 0.006), LAVI (OR = 1.04, 95% CI: 1.01–1.06, *P* = 0.001), S/D (OR = 0.27, 95% CI: 0.11–0.59, *P* = 0.002), and α (OR = 4.95, 95% CI: 2.05–12.79, *P* = 0.001)—found to be independently associated with LAT/SEC. Conversely, LVEF and NT-ProBNP did not independently predict SEC/LAT in NVAF patients. The results were shown in Table 2.

**Table 2:** Multivariate logistic regression analysis in the final model coefﬁcients.

Variables	β	SE	OR	95% CI	*Z*	*P*
Previous stroke/TIA	1.446	0.523	4.247	1.568–12.27	2.772	0.006
NT-proBNP, pg/ml, per 1,000 increase	0.248	0.174	1.282	0.922–1.844	1.424	0.154
LVEF	−0.027	0.019	0.974	0.937–1.010	−1.402	0.161
LAVI, ml/m^2^	0.034	0.012	1.035	1.014–1.057	3.276	0.001
S/D ratio	−1.320	0.416	0.267	0.114–0.589	−3.171	0.002
LA acceleration factor (α), per 0.1 increase	0.160	0.046	1.173	1.074–1.290	3.443	0.001

LA, left atrium; LAVI, left atrial volume index; LVEF, left ventricular ejection.

### 3.3 Construction and validation of the model

**Figure 5:**
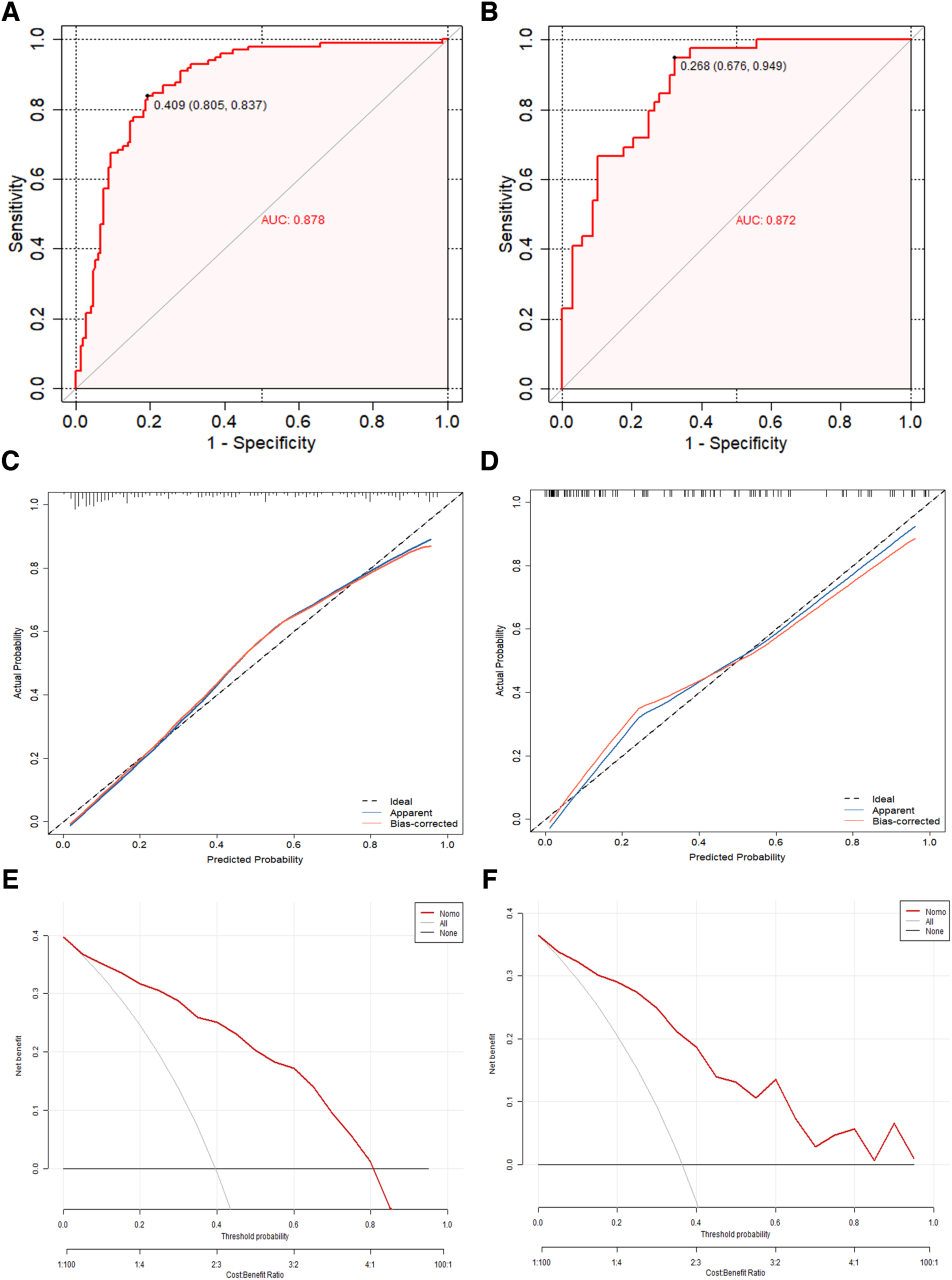
(**A,B)** The concordance index curve was used to evaluate the models’ ability to predict LAT/SEC in both the training cohort and validation cohort. (**C,D**) The calibration curves of the LAT/SEC risk nomogram were created by performing 500 bootstrap re-samples. The *y*-axis shows the number of confirmed cases of LAT/SEC risk, while the *x*-axis shows the predicted risk of LAT/SEC risk. The diagonal dotted line represents the predictions of an ideal model, and the solid line represents the performance of the actual data. The calibration plot closely aligned with the ideal 45° diagonal line, confirming the nomogram's accuracy in calibration. (**E,F**) Decision curve analysis was conducted for the nomogram in both the training cohort and the validation cohort. The decision curve of the nomogram consists of an *x*-axis that illustrates various possible thresholds for LAT/SEC risk and a *y*-axis that represents the net benefit achieved. (LAT/SEC, left atrial thrombus/spontaneous echo contrast).

By utilizing regression analysis, we constructed a nomogram incorporating the four significant variables (previous stroke/TIA, LAVI, α, and S/D) as components of the total score. This total score can be used to determine the probability of LAT/SEC in patients with NVAF by referring to the corresponding probability on the outcome axis (Figure 4C). Our nomogram demonstrated excellent discrimination efficiency with a C-statistic of 0.878 (Figure 5A). To further validate our findings, we performed internal validation using a bootstrap method with 500 repetitions. The calibration plot closely aligned with the ideal 45° diagonal line, confirming the accuracy of our nomogram's calibration (Figures 5C,D). With respect to validation cohort, our nomogram achieved a C-index of 0.872 for predicting the risk of LAT/SEC (Figure 5B). In the training cohort, the sensitivity and specificity values were found to be 83.7% and 80.5% respectively. Moreover, in the validation cohort, the sensitivity and specificity values were 94.9% and 64.6% respectively. For clinical application, we recommend using a cutoff of 128.0; patients exceeding this threshold are considered to be at a high risk for LAT/SEC.

Clinical decision curve analysis was performed to evaluate the model's clinical benefit. Both the training and validation groups demonstrated net benefits across a range of threshold probabilities. Both the model and the validation groups achieved net benefits when the threshold probability values were between 0.05 and 0.80, and between 0.02 and 0.85, respectively. Figures 5E,F illustrated the net benefit across these thresholds and demonstrated that the predictive model significantly enhanced risk stratification for LAT/SEC among NVAF patients.

**Figure 6:**
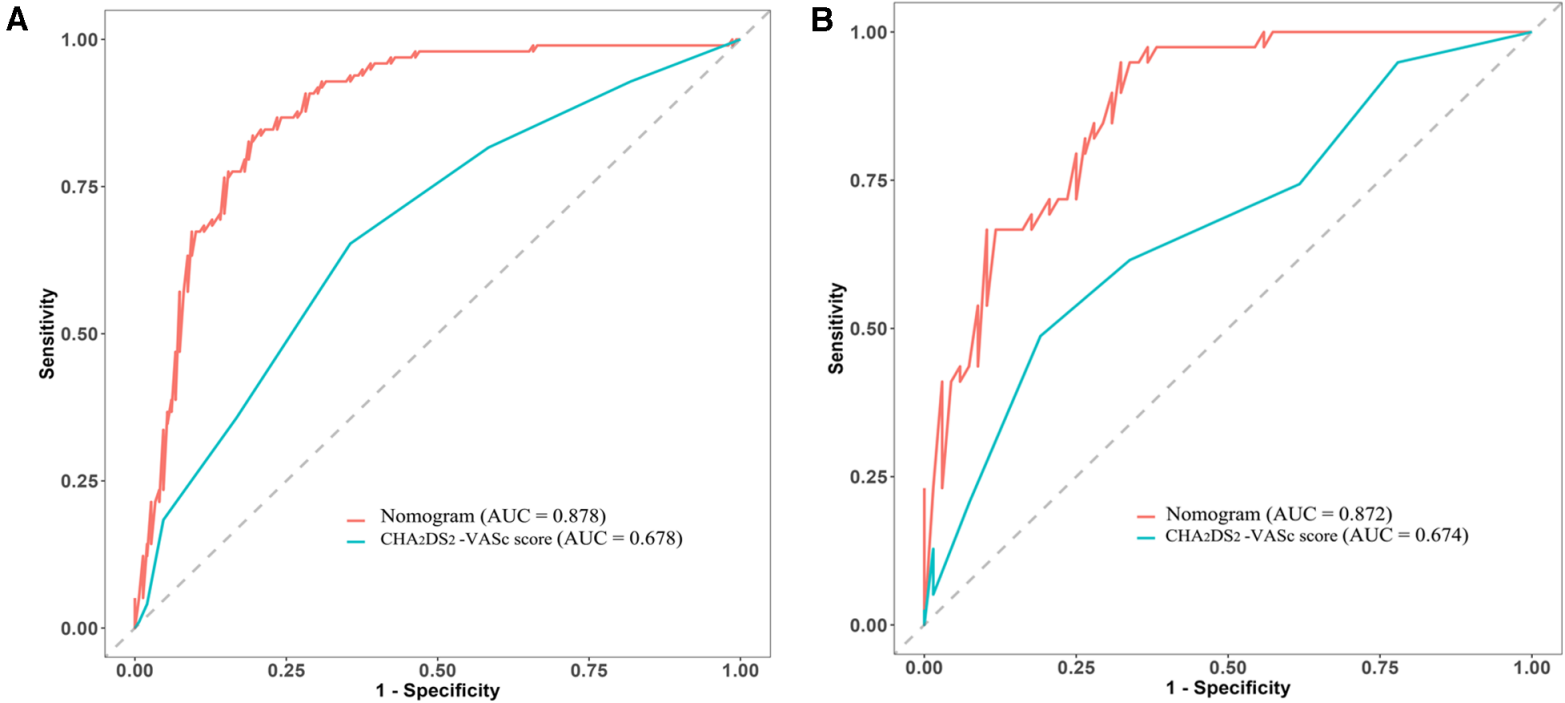
Comparative analysis of ROC curves between the nomogram and CHA2DS2-VASc scores in both the training cohort (**A**) and validation cohort (**B**).

Additionally, the ability of the CHA_2_DS_2_-VASc score to predict LAT/SEC was also examined via ROC curve analysis. The results revealed an AUC of 0.678 in the training cohort and 0.674 in the validation cohort. These findings suggest that the nomogram model exhibited superior predictive capabilities for LAT/SEC compared to the CHA_2_DS_2_-VASc score in both cohorts (Figure 6).

## 4 Discussion

The major findings of this study were as follows: (1) Assessment of left atrial hemodynamic parameters by transthoracic echocardiography, notably the left atrial acceleration factor (α) and the S/D ratio of the pulmonary vein, emerged as independent influencing factors of LAT/SEC in NVAF patients, beyond other risk factors and anticoagulation therapy. (2) The nomogram incorporating these these four variables (previous stroke/TIA, LAVI, S/D ratio, and α) could powerfully predict LAT/SEC risk and demonstrate the superior performance compared to CHA_2_DS_2_-VASc scores.

AF is widely recognized as a risk factor for stroke, and numerous studies have investigated the association between AF and stroke risk (12, 13). AF leads to disorganized atrial rhythms, creating vortices that can promote thrombus formation. This phenomenon is more pronounced in the LA, particularly in the left atrial appendage, due to its trabeculated surface, which results in slower blood flow. As a result, thrombosis in AF often originates from the left atrial appendage (14).

The CHA_2_DS_2_-VASc score is a commonly used clinical tool utilized for estimating the likelihood of stroke in patients with NVAF. It builds upon the CHADS_2_ score by considering additional risk factors, providing a more nuanced assessment of stroke risk. This score is often employed to determine whether anticoagulation therapy is necessary to prevent strokes in patients with NVAF. Previous studies have shown that the CHA_2_DS_2_-VASc score is effective in identifying patients at higher risk of stroke and thromboembolic events, regardless of whether they receive optimal anticoagulation therapy. Using this score allows clinicians accurately identify patients at truly high risk, enabling them to tailor individualized treatment strategies (15). However, the ability to distinguish risk for individual patients is limited, as indicated by C-statistic values ranging from 0.549 to 0.638 (16). This limitation has prompted the need to identify individuals at high risk of LAT/SEC, even if they have a low CHA_2_DS_2_-VASc score at baseline (17). These insights highlight the need for further investigation into stroke mechanisms in NVAF and the development of more refined risk stratification tools that incorporate both echocardiographic measurements and clinical parameters.

Several clinical conditions and biomarkers have been identified as independent influencing factors of stroke in AF patients (18, 19). Among these factors, LAT and SEC are significant markers of cardiogenic embolism in NVAF patients. Left atrial enlargement is strongly associated with LAT/SEC, which is an indicator of stroke risk (20). Numerous studies have definitively drawn connections between the dimensions of the LA and the incidence of ischemic stroke across diverse groups (21). Therefore, a nomogram that combines left atrial size with clinical risk factors could serve as a valuable predictor of LAT/SEC. However, the traditional measurement of left atrial diameter is relatively basic; future research using more precise echocardiography techniques could provide a better understanding of the relationship between stroke and both the size and function of the LA.

AF results in various hemodynamic changes that impact cardiac function and patient outcomes, such as alterations in atrial hemodynamics, ventricular response, and complications such as dementia. Understanding the hemodynamic changes in patients with AF can aid in the management of these patients. The primary mechanism behind thrombus generation in AF patients is the stagnation of blood within the LA. Previous research has suggested a notable correlation between LAT/SEC and LA hemodynamic parameters (22). Additionally, computational fluid dynamics analysis of the LA and left atrial appendage have shown potential value in improving stroke risk stratification. Fang et al. (23) demonstrated that hemodynamic details within these structures could enhance the accuracy of stroke risk in patients with AF.

Under normal physiological conditions, the pulmonary veins deliver oxygenated blood to the LA, which subsequently contracts in order to propel blood into the left ventricle. The LA pressure serves a dual role: driving blood through the trans-mitral outflow and resisting flow from the pulmonary veins into the atrium. The left atrial pressure is typically low and allows for the efficient filling of the left ventricle. Therefore, an increase in LA pressure may indicate faster outflows and reduced inflows. The LA acceleration factor (α), which quantifies the change in velocity of the LA during early diastole, has a strong relationship with pulmonary artery wedge pressure when evaluated by right heart catheterization (11). As it is well known, the pulmonary artery wedge pressure is approximately equivalent to the pressure in the LA. These findings imply that the α may serve as a non-invasive parameter for estimating the mean pulmonary artery wedge pressure. Elevated LA pressure can decrease blood flow velocities in the LA, particularly in its left atrial appendage, increasing the risk of stroke due to clot formation and abnormal blood flow. Importantly, the left atrial acceleration factor (α) may indicate dysfunction in patients with AF before anatomical enlargement occurs, making it a potential early marker of LA dysfunction. Research has also explored the relationship between the S/D ratio of pulmonary vein flow and stroke, suggesting its usefulness in assessing left ventricular diastolic function in AF patients (24). patients with AF and concurrent diastolic dysfunction are at a higher risk of developing atrial thrombus, which can lead to embolic strokes or other systemic embolic events. Consequently, a nomogram incorporating such hemodynamic parameters could greatly assist in stroke prevention in NVAF patients.

Our study synthesized traditional clinical factors, LA size and hemodynamic parameters to evaluate the risk of LAT/SEC. Utilizing LASSO regression and stepwise multivariate analysis, we identified several predictive factors, including previous stroke/TIA, LAVI, the S/D ratio, and left atrial acceleration factor (α). This nomogram model exhibited a remarkable ability to accurately identify high-risk individuals for LAT/SEC. These findings suggest that the addition of hemodynamic parameters to traditional factors improves the predictive power of risk for LAT/SEC in patients with NVAF, surpassing the discriminatory capability of CHA_2_DS_2_-VASc scores. In clinical settings, we propose a cutoff score of 128.0, with individuals exceeding this score regarded as high risk for LAT/SEC. This new model has the potential to replace traditional thrombosis assessments in patients with NVAF who are not eligible for TEE, and can provide guidance on the discontinuation of anticoagulants during follow-up. LA strain analysis is an advanced technique, in which speckle-tracking echocardiography is used to assess the deformation of the LA throughout the cardiac cycle. This technique provides important information about LA reservoir and conduit functions during AF. Patients with AF, especially those with impaired LA strain, are at a higher risk of thrombus formation due to abnormalities in atrial mechanics (25). Recent studies have indicated that evaluating LA strain can offer additional predictive value for determining the risk of LAT/SEC in patients with NVAF (26, 27). In future research, we will explore the additional value of utilizing traditional TTE parameters in combination with LA strain analysis to predict the risk of thrombosis in individuals with NVAF.

The current study has several limitations. Firstly, the nomogram model that was established using a cross-sectional design and had a limited sample size and lack of external validation. Therefore, further studies were needed to validate the accuracy and applicability of this predictive model. Secondly, our measurement of hemodynamic parameters was limited to a stable flow spectrum of mitral valve orifice and pulmonary veins in persistent AF patients; further complicated by the inherent challenges in accurately capturing pulmonary vein blood flow spectra, which are often affected by potential confounders like mitral regurgitation. Finally, LA or left atrial appendage LAT/SEC was used as an alternative indicator to evaluate the risk of stroke in NVAF patients. However, it should be noted that the correlation between SEC and thrombosis may vary depending on the grade of SEC, therefore the stroke risk with SEC is not completely equivalent to thrombosis.

## 5 Conclusion

In conclusion, our study has found that LA dilation, along with abnormal LA and pulmonary vein hemodynamics may associated with a higher risk of LAT/SEC. The hemodynamic changes of LA can be better understood by analyzing echocardiographic parameters, which provide valuable insights into the connection with LAT/SEC. Evaluating these hemodynamic parameters is crucial in predicting and stratifying stroke risk in NVAF patients. The association between LAT/SEC in NVAF patients is independently linked to previous stroke/TIA, LAVI, S/D ratio, and left atrial acceleration factor (α) as observed in this study. The nomogram combines these four variables to effectively predict the risk of LAT/SEC and shows better performance when compared to CHA_2_DS_2_-VASc score.

## Data Availability

The raw data supporting the conclusions of this article will be made available by the authors, without undue reservation.
